# Episodic angioedema with eosinophilia (Gleich's syndrome) associated with urticarial vasculitis: a coincidence or a novel clinical entity?

**DOI:** 10.1590/1414-431X202010745

**Published:** 2021-04-19

**Authors:** J.R. Basso, L.G.Z. Bizinoto, G.A. Limone, M.M.S.S. Enokihara, K. do Espirito-Santo, A.R. Fonseca, R.C. Agondi, A.F.T. de Gois, L.L. Cunha

**Affiliations:** 1 Departamento de Medicina, Escola Paulista de Medicina, Universidade Federal de São Paulo, São Paulo, SP, Brasil; 2 Departamento de Patologia, Escola Paulista de Medicina, Universidade Federal de São Paulo, São Paulo, SP, Brasil; 3 Disciplina de Hematologia, Escola Paulista de Medicina, Universidade Federal de São Paulo, São Paulo, SP, Brasil; 4 Serviço de Imunologia Clínica e Alergia do Hospital das Clínicas, Faculdade de Medicina, Universidade de São Paulo, São Paulo, SP, Brasil; 5 Disciplina da Medicina Baseada em Evidências, Escola Paulista de Medicina, Universidade Federal de São Paulo, São Paulo, SP, Brasil; 6 Programa de Pós-graduação em Endocrinologia e Metabolismo, Escola Paulista de Medicina, Universidade Federal de São Paulo, São Paulo, SP, Brasil

**Keywords:** Episodic angioedema with eosinophilia, Gleich's syndrome, Urticarial vasculitis

## Abstract

Episodic angioedema with eosinophilia (EAE) is a rare condition characterized by recurrent attacks of angioedema and urticaria accompanied by a marked elevation of peripheral eosinophil count. We report the case of a young female patient diagnosed with EAE associated with urticarial vasculitis. A 40-year-old female patient was admitted to our institution due to recurrent episodes of cheek and eyelid angioedema in the previous year. Episodes of facial angioedema lasted for two months with spontaneous remission afterwards. In addition, she presented pruritic and painful skin eruptions of erythematous circles, which persisted for longer than 24 h, that were palpable, somewhat purplish, and more pronounced on the face, arms, and trunk. Laboratory investigation showed a sustained elevation of white cell counts with marked eosinophilia. Serum IgM, IgE, and IgA were normal; IgG was slightly elevated. C1-esterase inhibitor and tryptase test were normal. Reverse transcriptase-polymerase chain reaction was performed for detection of *FIP1L1-PDGFRA* and *BCR-ABL* rearrangements. None of these alterations were found. Skin biopsies were suggestive of urticarial vasculitis. The patient was submitted to esophagogastroduodenoscopy, which showed mild chronic gastritis, with no eosinophilic infiltration. Cardiac dimensions and function were normal. Abdominal ultrasound and total body CT-scan failed to show lymphadenopathy, organomegaly, and tumors. We report the first case of association between episodic angioedema with eosinophilia and urticarial vasculitis. It is possible that both conditions share a physiopathological mechanism, suggesting that it is not just a chance association.

## Introduction

Eosinophilia is a common finding in routine tests that is defined as a blood eosinophil count exceeding 500 cells/mm^3^ (1). The differential diagnosis may include many underlying conditions (2). Even though some algorithms have been proposed, the evaluation of eosinophilia still poses a challenge in clinical practice (3). One possible diagnosis is the episodic angioedema with eosinophilia (EAE). EAE was firstly described by Gleich et al. (4). Since then, nearly one hundred cases have been reported (5). EAE is a rare condition characterized by recurrent attacks of angioedema and urticaria accompanied by a marked elevation of peripheral eosinophil account. Herein, we report the case of a young female patient diagnosed with EAE associated with hypocomplementemic urticarial vasculitis.

## Case report

**Figure 1:**
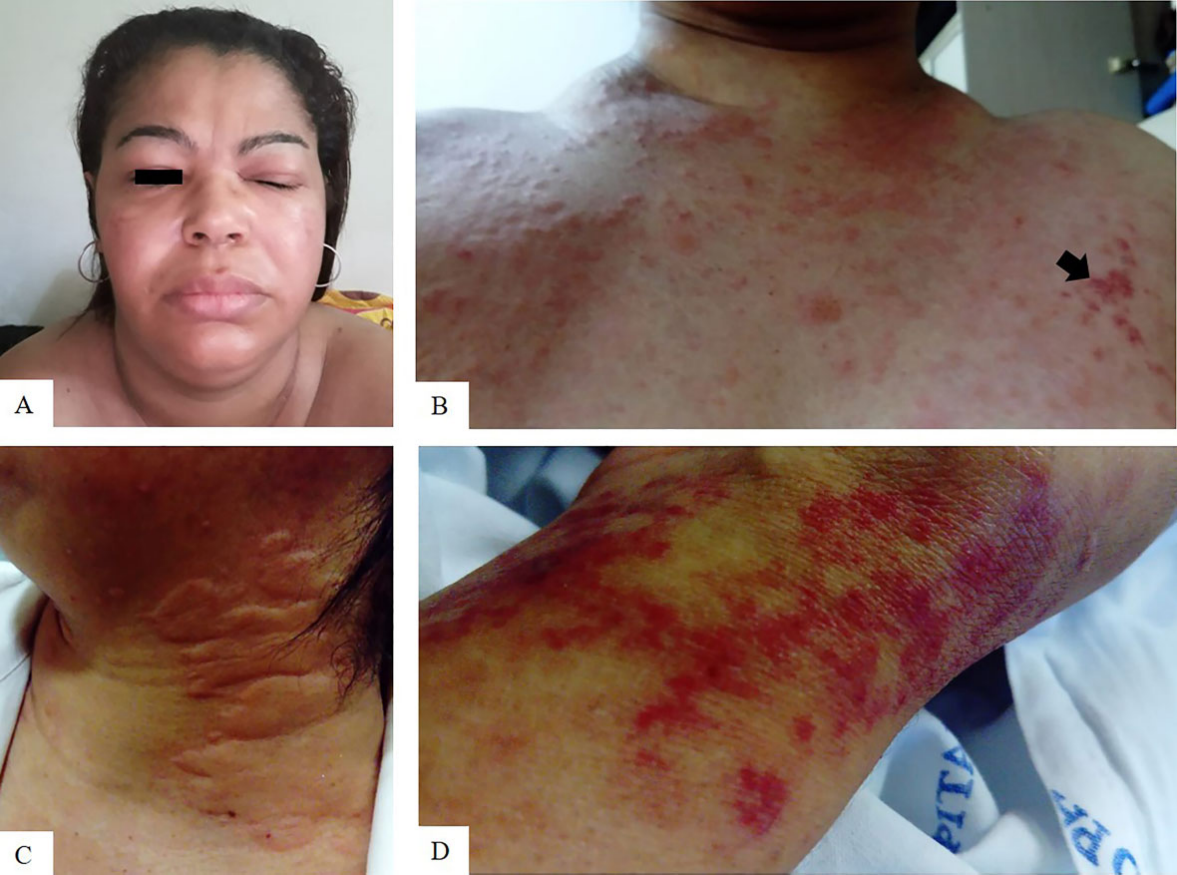
**A**, Asymmetrical facial angioedema. Angioedema was more prominent on the cheek and eyelid. **B**, Presence of hives on the thorax. Pruritic and painful purplish papules were observed on the left anterior thorax (black arrow). **C**, Urticarial circles that emerged on the lateral neck. **D**, Painful purplish macules can be seen, suggesting vasculitis.

A 40-year-old female patient was admitted to our institution (Internal Medicine Outpatient Unit, Federal University of São Paulo, Brazil) due to recurrent episodes of cheek and eyelid angioedema in the previous year. Episodes of facial angioedema lasted for two months with spontaneous remission. No evident allergic trigger was identified. The episodic angioedema was added to pruritic and painful skin eruption of erythematous circles, which persisted for more than 24 h, that were palpable, somewhat purplish, and more pronounced on the face, arms, and trunk (Figure 1A-D). At admission, the patient denied any fever, myalgia, arthralgia, and weight loss. No family history of similar findings was reported. Physical examination indicated neither arthritis nor mucosal ulceration. Appropriate consent and permission for publication were obtained.

**Figure 2:**
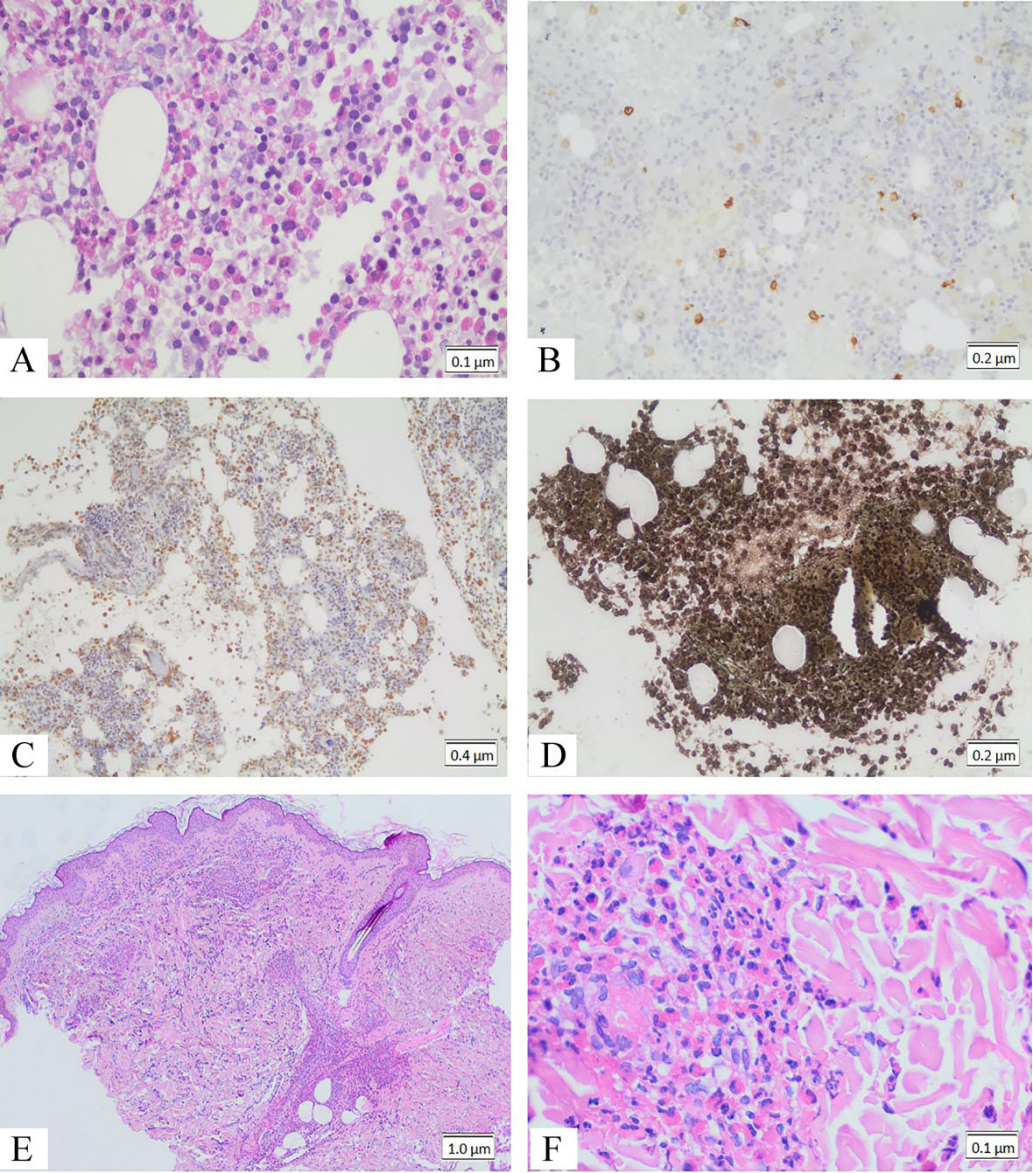
**A**, Bone marrow biopsy (HE 400×, scale bar 0.1 μm) with an enrichment of eosinophils. **B**, Bone marrow biopsy (200×, scale bar 0.2 μm) with CD117 immunostaining, suggesting an increase of mast cells. The immunostaining for myeloperoxidase (**C**, 100×, scale bar 0.4 μm) shows an elevated myeloid to erythroid proportion. Bone marrow presented grade 1 fibrosis, as evidenced by reticulin staining (**D**, 200×, scale bar 0.2 μm). Panels **E** (40×, scale bar 1 μm) and **F** (400×, scale bar 0.1 μm), Skin biopsy showing dermal small capillary vessels with inflammatory infiltrate in the walls, suggesting urticarial vasculitis.

**Figure 3:**
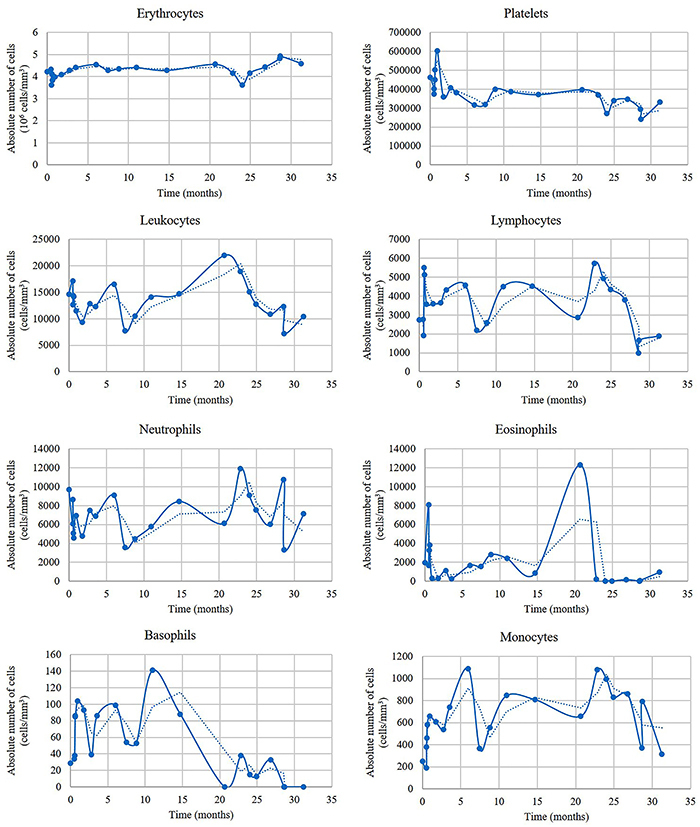
Serial blood cell counts after the patient began treatment. Dashed lines represent the simple moving average trend. Leukocyte counts, especially neutrophils and eosinophils, present a sinusoidal-like trend, suggesting a cyclic pattern. One could think that the cyclic shape could be due to irregular adherence of the patient to corticoid-based treatment. Another explanation could be that the multilineage cell cycling observed in patients with episodic angioedema with eosinophilia occurs despite corticoid treatment.

Laboratory investigation showed a sustained elevation of white cell count (∼17,100 cells/mm^3^) with marked eosinophilia (∼8100 cells/mm^3^). No abnormal blood cell was found in the blood smear. Kidney and liver function, folic acid, and vitamin B12 were normal. Urine sediment was normal. Serological tests failed to show infection by common agents such as HIV, syphilis, viral hepatitis, cytomegalovirus, Epstein-Barr virus, and *Toxoplasma spp*. No parasitic diseases were identified. Erythrocyte sedimentation rate was 32 mm/hr (NR <20 mm/hr) and rheumatoid factor was negative. Cryoglobulins, antinuclear antibodies, antineutrophil cytoplasmic antibodies, and extractable nuclear antigen antibodies panel were negative. C3 and C4 complement proteins were undetected. Serum protein electrophoresis found a discrete non-clonal elevation of gamma-globulin zone (2.03 g/dL, NR 0.7-1.5 g/dL). Serum IgM was 116 mg/dL (NR 40-230 mg/dL) and IgE and IgA were normal as well; IgG was slightly elevated (1905 mg/dL, NR 700-1600 mg/dL). C1-esterase inhibitor (quantitative and functional) and tryptase tests were normal.

Histologic sections of bone marrow biopsy (Figure 2A-D) showed a high cellularity specimen (around 70%), with increased myeloid cells, markedly exhibiting eosinophilic differentiation, leading to 8:1 granulocyte to erythroid proportion. The megakaryocyte elements were increased in number, without maturation disturbance. There was slight reticulin deposition. RNA was extracted from peripheral white blood cells. Reverse transcriptase-polymerase chain reaction was performed for detection of *FIP1L1-PDGFRA* and *BCR-ABL* rearrangements. The *FIP1L1-PDGFRA* rearrangement is associated with chronic eosinophilic leukemia and is frequently found in a subset of patients with systemic mastocytosis who also have eosinophilia (6). The presence of the *FIP1L1-PDGFRA* rearrangement guides the treatment of these conditions. The *BCR/ABL* rearrangement is associated with acute lymphoblastic leukemia and chronic myeloid leukemia (7). The identification of the *BCR/ABL* rearrangement has an important prognostic value for patients with acute lymphoblastic leukemia and can be used as therapeutic monitoring for patients with chronic myeloid leukemia. None of these alterations was found.

Skin biopsies (Figure 2E and F) showed a leukocytoclastic vasculitis, predominantly with intact and degenerated neutrophils and eosinophils. The histologic findings were suggestive of urticarial vasculitis. In addition, we observed a residual interface dermatitis, with thickening of the basal membrane and dermal intracellular mucin deposition.

The patient was submitted to esophagogastroduodenoscopy, which showed a mild chronic gastritis, with no eosinophilic infiltration. Echocardiographic study showed that cardiac dimensions and function were normal. Abdominal ultrasound and total body CT-scan failed to show lymphadenopathy, organomegaly, and tumors.

The patient did not respond to hydroxychloroquine, methotrexate, or antihistamines. Gathering clinical, laboratory, and molecular data, she was diagnosed with EAE associated with hypocomplementemic urticarial vasculitis. We opted for a prednisone tapering strategy. The initial dose was 1 mg/kg per day and the dose was decreased by 10 mg each following week. We decreased by 2.5-mg increments until 20 mg/day was reached. In addition, we prescribed dapsone (100 mg/day) in order to spare corticoid use during follow-up. The patient reached remission after 4 weeks of treatment. An attempt to discontinue prednisone was done. However, the patient presented a relapse. We opted to use a corticoid tapering strategy again. Finally, a continuous dose of prednisone (5 mg/day) and dapsone (100 mg/day) was maintained and the patient obtained remission. The patient has been followed-up and consecutive blood cell counts have been taken (Figure 3). She is currently asymptomatic with 5 mg/day prednisone for 2 years.

This patient presented EAE concurrent with urticarial vasculitis, both considered to be uncommon diseases, and no other constitutional symptom. EAE is more prevalent among young women, and in general, constitutional symptoms such as fever or weight loss are not observed in the majority of subjects described in the literature (8). Compared with the literature, our patient presented marked eosinophilia (∼8100 cells/mm^3^). A wide range of total eosinophil counts can be found at presentation (from 1000 to 23,400 cells/mm^3^) (8). In contrast to hypereosinophilic syndromes, organ dysfunction is not observed among patients with EAE (9). Indeed, an extensive diagnostic workup failed to find eosinophilic infiltration and organ dysfunction in our patient. Increased IgM and eosinophilia are ordinary findings among patients with EAE. However, as described in a previous series (8), our patient did not present relevant elevation of IgM.

Skin biopsy showed concurrent urticarial vasculitis. The pathophysiology of both EAE and urticarial vasculitis is still unclear. Khoury et al. observed that patients with EAE present cyclic variations of eosinophil, mast cell, neutrophil, and lymphocyte counts (10). The expansion of these cells follows the elevation of interleukin (IL)-5, IL-13, IL-9, and IL-10 serum levels, suggesting that type 2 cytokines may be involved in the pathogenesis of EAE (10). We observed that the bone marrow niche of the patient was hypercellular and enriched with eosinophils and mast cells precursors, reinforcing that an orchestration of T helper 2 (Th2) immune response may be elicited in EAE. It is possible that the expansion of eosinophil and mast cells reverberates to the peripheral blood, leading to vascular injury and vasculitis. Skin biopsy of our patient showed both eosinophils and neutrophils in vascular injury. In fact, patients with urticarial vasculitis present a hallmark perineural, perivascular, and interstitial infiltration of eosinophils, indicating that peripheral eosinophils may contribute to the pathogenesis of urticarial vasculitis (11).

To our knowledge, this is the first report of the association between EAE and urticarial vasculitis. Whether there is a causative link or it is just a serendipitous pathological finding remains to be elucidated. More studies are warranted to further investigate the pathogenic link between EAE and urticarial vasculitis.

## References

[1] Roufosse F (2015). Management of hypereosinophilic syndromes. Immunol Allergy Clin North Am.

[2] Simon HU, Rothenberg ME, Bochner BS, Weller PF, Wardlaw AJ, Wechsler ME (2010). Refining the definition of hypereosinophilic syndrome. J Allergy Clin Immunol.

[3] Leru PM (2019). Eosinophilic disorders: evaluation of current classification and diagnostic criteria, proposal of a practical diagnostic algorithm. Clin Transl Allergy.

[4] Gleich GJ, Schroeter AL, Marcoux JP, Sachs MI, O'Connell EJ, Kohler PF (1984). Episodic angioedema associated with eosinophilia. N Engl J Med.

[5] Bertrand V, Boccara O, Filhon B, Manca F, Lefàvre G, Groh M (2020). Episodic angioedema with eosinophilia (Gleich syndrome) in children: a clinical review. Pediatr Allergy Immunol.

[6] Curtis C, Ogbogu P (2016). Hypereosinophilic syndrome. Clin Rev Allergy Immunol.

[7] Jabbour E, Kantarjian H (2018). Chronic myeloid leukemia: 2018 update on diagnosis, therapy and monitoring. Am J Hematol.

[8] Cho HJ, Yoo HS, Kim MA, Shin YS, Ye YM, Nahm DH (2014). Clinical characteristics of angioedema with eosinophilia. Allergy Asthma Immunol Res.

[9] Liu F, Hu W, Liu H, Zhang M, Sang H (2017). Episodic angioedema associated with eosinophilia. An Bras Dermatol.

[10] Khoury P, Herold J, Alpaugh A, Dinerman E, Holland-Thomas N, Stoddard J (2015). Episodic angioedema with eosinophilia (Gleich syndrome) is a multilineage cell cycling disorder. Haematologica.

[11] Kamyab K, Ghodsi SZ, Ghanadan A, Taghizadeh J, Karimi S, Nasimi M (2019). Eosinophilic infiltration: an under-reported histological finding in urticarial vasculitis. Int J Dermatol.

